# Identification and Structural Characterization of Viroporins from Deadly Hemorrhagic Viruses

**DOI:** 10.3390/v17081120

**Published:** 2025-08-14

**Authors:** Hiya Lahiri, Kingshuk Basu, Isaiah T. Arkin

**Affiliations:** 1 Department of Biological Chemistry, The Alexander Silberman Institute of Life Sciences, The Hebrew University of Jerusalem, Edmond J. Safra Campus, Jerusalem 9190400, Israel; hiya.lahiri@mail.huji.ac.il; 2 Department of Biomedical Engineering, City University of Hong Kong, Tat Chee Avenue, Kowloon, Hong Kong, China; kingbasu@cityu.edu.hk

**Keywords:** Crimean–Congo hemorrhagic fever, Ebola virus disease, viroporins, structural analysis, ion channels

## Abstract

Crimean–Congo hemorrhagic fever virus (CCHF-V) and Ebola virus are lethal pathogens that cause widespread outbreaks of hemorrhagic fever. Both diseases can be transmitted through contact with the bodily fluids of infected individuals, but as an arbovirus, CCHF-V is primarily transmitted through tick bites. Both of these viruses are classified as Risk Group 4 due to the appreciable health threat they pose. To date, there are few effective treatments available to combat these deadly hemorrhagic fevers. Consequently, identifying and characterizing ion channels (viroporins) encoded in the viral genomes may lead to potential targeted drug development. Therefore, using bacteria-based genetic assays, two viroporin candidates from CCHF-V and Ebola have been examined, and their proposed structures have been modeled to aid in further drug discovery. The results indicate that CCHF-V-gp exhibits channel activity, which is indistinguishable from established viroporins found in other viruses. In contrast, our experimental approach was unable to uncover a viroporin candidate in the Ebola virus.

## 1. Introduction

Crimean–Congo hemorrhagic fever virus (CCHF-V) and Ebola virus are two deadly pathogenic viruses that cause severe hemorrhagic fevers in humans. CCHF has a high mortality rate of 40% worldwide and is found in Asia, Africa, and many parts of Europe [1,2,3,4]. The etiological agent of CCHF is the eponymous CCHF-V that belongs to the genus *Orthonairovirus*, family *Nairoviridae*, and class *Bunyaviricetes* [5]. The CCHF virus’s genome is made up of three segments of negative-strand RNA: The small segment encodes the nucleoprotein, while the medium segment encodes the glycoprotein precursor that eventually is spliced into several other mature proteins. The glycoprotein of the CCHF-V is responsible for determining the virus’ preference to infect specific host types and also influences the highly pathogenicity of the virus when infecting humans [6]. The glycoprotein encoded by the M gene is considered to play multiple roles including viral entry, packaging, tropism, and pathogenicity. In nairoviruses, the gp processing is a complicated process, where the mature glycoprotein produces Gn, Gc, and several other nonstructural secreted glycoproteins [7]. Finally, the large segment produces the L protein that contains the RNA-dependent RNA polymerase [8,9,10]. CCHF-V primarily spreads through arthropods such as ticks and flies. Additionally, it can be transmitted through direct contact with body fluids and tissue from an infected individual [11].

Due to the high mortality of CCHF, higher transmission rates can lead to a public health disaster. Without any specific antiviral drugs available, fast detection and supportive therapy are the only options for treating CCHF. Ribavirin, a broad-spectrum antiviral, has been used to treat CCHF, but the therapeutic advantages have not been well observed [12]. Consequently, the need for proper antiviral drug development commands the highest priority.

Ebola disease is caused by the Ebola virus, which belongs to the *Orthoebolavirus* genus, family *Filoviridae*, and order *Mononegavirales*. Similar to CCHF-V, it leads to severe illness that can result in death. Four species of Ebola viruses are known to cause severe disease in humans: Ebola virus (Zaire), Sudan Ebola virus, Taï Forest Ebola virus, and Bundibugyo virus. In contrast, Reston Ebola virus primarily affects primates [13]. These filoviruses are enveloped and filamentous and have a non-segmented linear negative-sense RNA genome [14]. The 19-kilobase-long genome encodes nine translational products that include the viral nucleoprotein (NP), membrane glycoprotein, viral protein VP35, VP40, VP30, VP24, and large protein L [15]. The viral glycoprotein gene has three overlapping ORFs; it produces the pre-protein named pre-sGP(soluble secreted glycoprotein). pre-sGP is ultimately cleaved to produce sGP and the carboxy-terminal delta peptide [16]. It was previously observed that the delta peptide can permeabilize the cell membrane and can increase the ion permeability of the host cell [17]. Another study showed that the Ebola delta peptide acts as an enterotoxin in the host cell [18]. A severe outbreak of Ebola virus disease was observed in West Africa from 2013 to 2016, resulting in more than 28,000 cases, which led to nearly 50% mortality [19]. The initial care required for Ebola disease is optimized supportive care, as defined by the World Health Organization (WHO) in its guidelines [20].

In contrast to CCHF, where no vaccine is available, vaccines exhibiting high effectiveness against Ebola virus (Zaire) have been approved in the last decade [21], with several more under development [22]. The success of vaccine development led to the development of monoclonal antibodies against Ebola virus disease stemming from convalescent plasma therapy: mAb114 (ansuvimab [23] or REGN-EB3 Inmazeb™ [24]). Ansuvimab, also known as EBANGA™, was derived from a monoclonal antibody from an Ebola disease survivor, and it targets the Ebola virus surface glycoprotein by impairing the viral entry [25]. Inmazeb is a combination of three immunoglobulins that individually target distinct glycoprotein epitopes, which leads to neutralizing the virus and increasing antibody-dependent cellular cytotoxicity (ADCC) [24]. However, at present, there are no small-molecule drugs against Ebola, exemplifying the need to develop targets that can serve as the subject of new antiviral agents.

A potential target for antiviral drug development is viral ion channels [26,27,28,29]. Many viruses are known to encode such channels in their genome that are collectively coined viroporins. They are generally small in size, as exemplified by the archetypical 96-amino acids M2 from the Influenza A virus that forms an H^+^ channel [30]. M2 was shown to serve as the target of aminoadamantanes [31], the second antiviral drug approved for use [32]. Regrettably, wide-scale resistance has vitiated the utility of aminoadamantanes [33,34]. Since channels as a family are excellent drug targets, viral ion channels may represent an attractive target for antiviral drug development. Viroporins have several functional activities that include viral replication, assembly, viral entry, and release from the host cells. Apart from the Influenza A M2, there are several other viruses whose viroporins [35] have been characterized, for example, the hepatitis C virus [36], coronavirus [37], arenavirus [38], and togaviruses [39]. Recently, Harrison et al. have quantitatively assayed the number of ion channels present in SARS-CoV-2 using electrophysiology [40].

To date, there are no known viroporins available for targeting in CCHF-V. Keeping that in mind, herein, we identified and characterized one glycoprotein (gp) from CCHF-V by bacteria-based genetic assays, which can form a potential ion channel in the host cell. We also provide a probable molecular structure of the protein using computational analyses. For the Ebola virus (Zaire), there is a report that a 40-residue segment (delta peptide) within the C-terminal end of the small secreted glycoprotein (sGP) can serve as a viroporin [17]. Consequently, we examined the ion channel activity of the delta peptide by the same bacteria-based assays, alongside molecular modeling. Comparative analysis of the results of the bacteria-based experimental assays and computational analyses provided insight into the potential of each of these proteins to serve as a target for developing antiviral channel blockers.

## 2. Materials and Methods

### 2.1. Cloning

gBlocks of the CCHF-V-gp (ACK58347.1) [41] and Zaire Ebola virus delta peptide (ASV62183.1) [42], which can serve as potential viroporins, were constructed with the desired sequence along with a spacer, a histidine tag, and a stop codon at the C-terminal and were ordered from Integrated DNA Technologies (Coralville, IA, USA). All the proteins were then expressed as a fusion protein with maltose-binding protein (MBP) using the pMAL-p2X plasmid (New England BioLabs; Ipswich, MA, USA) and the Gibson Assembly^®^ Cloning Kit (New England BioLabs).

### 2.2. Bacterial Strain

DH10B, a K12 *Escherichia coli* strain, was purchased from Invitrogen (Carlsbad, CA, USA); LB650 potassium uptake deficit strain (Δ*trkG*, Δ*trkH*, and Δ*kdpABC5*) [43] was a kind gift from Professor K. Jung, Ludwig-Maximilians Universität München, and Professor G.A. Berkowitz, University of Connecticut, and the LR1 strain for fluorescence-pH assay was kindly donated by Professors M. Willemoës and K. Lindorff-Larsen, Københavns Universitet.

### 2.3. Chemicals

All the chemicals were purchased from Sigma-Aldrich Laboratories (Rehovot, Israel), with the exception of Isopropyl-β-D-1-thiogalactopyranoside (IPTG), which was purchased from Biochemika-Fluka (Buchs, Switzerland).

### 2.4. Bacteria Based Assays

Three bacteria-based channel assays: negative, positive, and fluorescence-pH, were used to examine the channel activity of the proteins in question. Below, we expound on the details of each method.

#### 2.4.1. Negative Assay

DH10B cells were grown overnight in Lysogeny Broth (LB) at 37 °C, diluted 500-fold, and grown until they reached an OD600 of 0.2. Then, 50 μL of the desired solution, along with another 50 μL of this cell culture, was added to a 96-well plate. Different concentrations of Isopropyl-β-D-1-thiogalactopyranoside (IPTG) were added along with 1% D-glucose, and controls were taken without IPTG induction. The plate was incubated in a multiplate reader (LogPhase 600 from BioTek; Santa Clara, CA, USA) at 37 °C for 16 h. Readings were taken at 15 min time intervals and plotted over time. Duplicate or triplicate sets were conducted for each experiment.

#### 2.4.2. Positive Assay

LB650 bacteria were grown overnight by following the same protocol, except for the fact that the LB media were replaced by LBK media that contained 150 mM KCl instead of NaCl. Prior to protein induction, the media were replaced with LB.

#### 2.4.3. Fluorescence-pH Assay

The LR1 bacterial strain was used for this assay, whose chromosomal DNA encodes for a pH-sensitive green fluorescent protein (GFP) [44]. These LR1 cells with different viroporin chimeras were grown overnight in LB media containing 1% glucose. At 1:500 ratio, the overnight-grown culture was diluted and grown until the OD600 reached 0.6 to 0.8. Different concentrations of IPTG were used for the induction, and then all were diluted to reach an OD600 of 0.2. Cultures without IPTG were used as a control set. After an OD600 of 0.2 was reached, cells were pelleted down by centrifuging at 3000× *g* for 10 min. The cells were then washed and dissolved in McILvaine Buffer [45]: 200 mM Na2HPO4 and 0.9% NaCl adjusted to pH 7.6 with 0.1 M citric acid. Then, to each well of a 96-well plate (Nunclon f96 Microwell Black Polystyrene, Thermo Fisher Scientific; Waltham, MA, USA), 30 μL of McILvaine buffer were added along with 200 μL of culture. Three wells contained McILvaine buffer and bacterial cultures but without induction, which were used as the control. Then, 70 μL of citric acid (300 mM, 0.9% NaCl) was added at the starting point to each well by a liquid handling system (Tecan; Männedorf, Switzerland). The fluorescent measurements were carried out at an ambient temperature in a microplate reader (Infinite F200 Pro, Tecan) recording emissions at 520 nm and excitation at 390 or 466 nm. The proton concentrations were then finally calculated using the ratio of the two wavelengths as described [44].

#### 2.4.4. Computational Studies

Primary protein sequences (oligomeric) were fed into AlphaFold2.ipynb using ColabFold v1.5.2: AlphaFold2 to obtain tentative three dimensional structures [46]. The proteins were subsequently embedded within a pre-equilibrated ER–Golgi intermediate compartment (ERGIC) membrane composed of 1-palmitoyl-2-oleoyl-sn-glycero-3-phosphoethanolamine (POPE), 1-palmitoyl-2-oleoyl-sn-glycero-3-phosphocholine (POPC), and 1-palmitoyl-2-oleoyl-sn-glycero-3-phospho-L-serine (POPS) in a 3:1:1 ratio using the CHARMM-GUI [47,48,49]. Position-restraints (100,000 kcal/mol/${Å}^{2}$) on protein-heavy atoms were used to ensure that the protein did not change during energy minimization, which was accomplished using the steepest descent minimization algorithm with a tolerance of 500 kJ mol^−1^ nm^−1^.

Molecular dynamics (MD) simulations were run for 100 ns using Gromacs version 2024.4 [50,51,52,53,54] employing the CHARMM36m force field [55]. The LINCS algorithm with an integration time step of 2 fs was used in all cases to constrain the length and angles of the hydrogen atoms [56]. The atomic coordinates were saved at every 500 ps interval. The temperature for reference was set at 323 K, and the solvent, lipids, and proteins were coupled separately into a Nosé–Hoover temperature bath [57,58], with a coupling constant value of τ=0.5 ps. The pressure coupling was obtained with a Parrinello–Rahman barostat with τ=2 ps [59,60]. A 1.2 nm cut-off was set for van der Waals interactions. The frequency of updating the neighbor list was kept at 10 fs. The electrostatic parameters were calculated using fourth order Particle Mesh Ewald (PME) long-range electrostatics [61], and the cut-off short-range electrostatics was set to 1.2 nm. All the simulation boxes contained 190 lipid molecules and 19,481 water molecules approximately, with Na^+^ and Cl^−^ counter ions (at 0.15 M concentration). Water molecules were fitted using the FLEXSPC model [62], and the total number of atoms was in the 86,000 range. The root mean square deviation (RMSD), and root mean square fluctuations (RMSF), values as a function of time were extracted. Structures of the simulated proteins were visualized and analyzed with VMD software version 1.9.4a51 [63].

## 3. Results

Potential viroporins were selected by screening the viral genome open reading frames utilizing the characteristic features of viroporins: short length (<100 amino acids) with one or more transmembrane (TM) domains [26,27,28,29], as exemplified by the archetypical influenza A M2 [30,64,65]. In the case of CCHF-V, a 91 amino acid glycoprotein (GenBank: ACK58347.1 [41]) was found to contain a single TM domain as confirmed by TMHMM2.0 [66], making it a potential candidate for the search of ion-channel activity. However, for the Zaire Ebola virus, no candidate was found with the aforementioned characteristics. In particular, two proteins with TM domains were identified, but they were significantly larger than what is commonly found in viroporins [26,27,28,29]: secreted glycoprotein and the spike glycoprotein with 364 and 670 amino acids, respectively. However, it was previously reported that the 40 amino acid delta peptide (NCBI number ASV62183.1 [42]) can serve as a viroporin [17], despite lacking an identifiable TM domain. Taken together, we examined the channel activities of both proteins as expounded below.

### 3.1. Channel Activity in a Bacterial-Based Assay

The channel activity of CCHF-V and Zaire Ebola virus proteins was assessed using three bacteria-based channel assays. In these assays, the activities of each protein are reflected in the phenotypical changes of the bacterial strains. In all assays, the viral proteins were fused to the carboxy-terminus of the maltose-binding protein (MBP) to ensure efficient expression and transport to the periplasmic space. If the protein contains a TM domain, it would halt the transport and anchor the protein in the inner membrane of the bacteria. Finally, due to their small size and simplicity, this chimeric construct has proven applicable to numerous varied viroporins [67,68,69,70,71,72,73].

#### 3.1.1. Negative Assay

**Figure 1:**
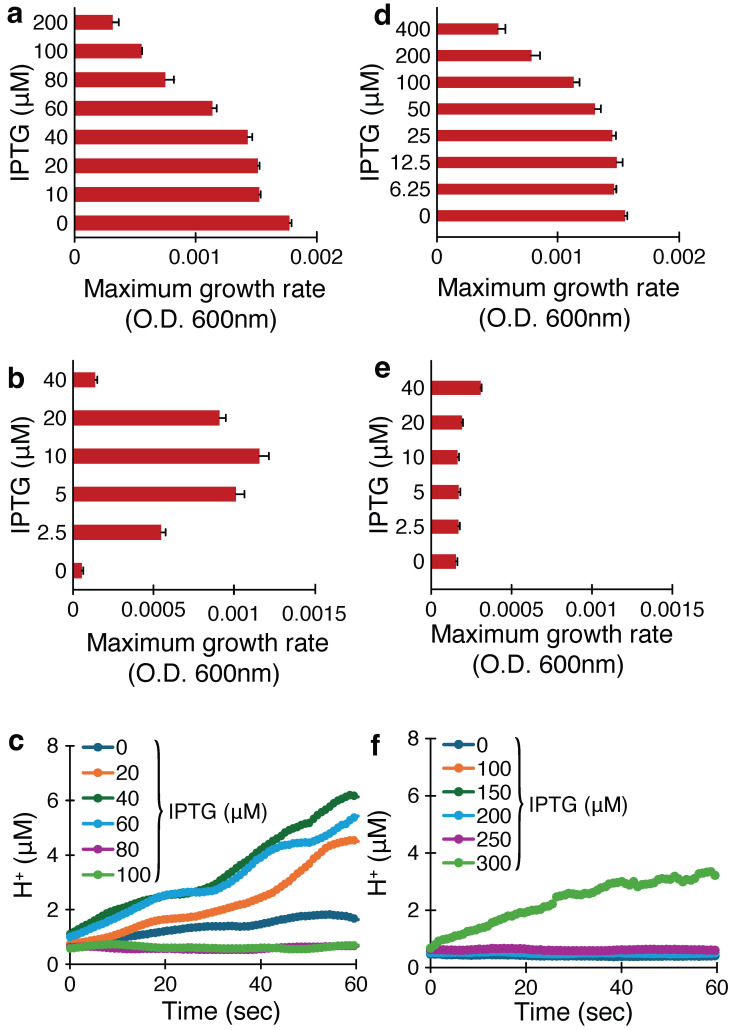
Three bacteria-based assays to asses the channel activity of CCHF-V-gp protein (**a**–**c**) and Ebola virus delta peptide (**d**–**f**): negative assay panels (**a**,**d**); positive assay panels (**b**,**e**); fluorescence-pH assay panels (**c**,**f**).

In this assay, the protein is expressed in the bacterial inner membrane at increasing levels, which causes an increase in permeability that ultimately is reflected by a decrease in bacterial growth [67]. Figure 1a shows that the growth of bacteria is reduced upon induction with Isopropyl-β-D-1-thiogalactopyranoside (IPTG) that leads to expression of CCHF-V-gp. Upon increasing the concentration of the inducer from 10 to 100 μM, the bacterial growth is reduced gradually, which is plotted against the inducer concentration versus bacterial growth (OD600). In particular, at 80 μM of IPTG, the bacterial growth is roughly halved.

Results from a similar analysis of the Ebola virus delta peptide are depicted in Figure 1d. In this instance, it can be seen that higher concentrations of the inducer are required to reduce bacterial growth, whereby 200 μM of IPTG halves the bacterial growth.

#### 3.1.2. Positive Assay

This assay is the reciprocal to the negative assay described above: K^+^-uptake deficient bacteria can only grow when the media are supplemented by potassium or when they express a channel that can transport potassium [43,74]. Figure 1b and Figure 1e show the positive assay results for the gp of CCHF-V and delta peptide from Ebola virus, respectively. For the CCHF-V-gp, a significant rise in bacterial growth is seen upon increasing the inducer concentration up to 10 μM. At elevated inducer concentration (≥20 μM), the growth rate decreases. In contrast, the Ebola virus delta peptide performs poorly in the positive assay: a much larger concentration of inducer (40 μM) is required to obtain a minor growth rate enhancement.

#### 3.1.3. Fluorescence-pH Assay

Following the above complementary assays, a final assay was performed where the expression of the viroporin impacts H^+^ uptake into the bacteria. For this assay, we expressed the viral protein in bacterial cells that constitutively express a pH-sensitive green fluorescence protein (GFP), which has two excitation maxima at 390 nm and 466 nm, and their ratio of these is a function of pH [75]. Consequently, H^+^ transport through the expressed viral protein can be monitored by a change in fluorescence. Figure 1c and Figure 1f show the change in H^+^ concentration upon expression of CCHF-V gp and Zaire Ebola virus’ delta peptide, respectively.

Once again, a clear picture is obtained whereby the expression of the CCHF-V gp leads to the acidification of the bacterial cytoplasm due to significant H^+^ flux: after one minute, 20 μM of IPTG causes the cytoplasmic [H^+^] to approach 5 μM. Note that the elevated inducer concentrations beyond 60 μM result in decreased acidification. In contrast, Zaire Ebola virus’ delta peptide leads to minor acidification (ca. [H^+^] 3 μM) only when the inducer concentration is raised considerably to 300 μM.

### 3.2. Structural Analysis and Molecular Modeling

Using TMHMM2.0 for CCHF-V-gp, a 22-amino-acid-long transmembrane sequence was found between Gly5 and Leu27. Segment 28 to 91 contains 8 arginine and 11 lysine residues carrying positive charges, which are only partially counterbalanced by five aspartic and five glutamic acid residues. This charge distribution provides an overall positive charge and makes it the probable cytosolic end of the channel [76]. Starting with this initial topological prediction, we attempted to derive a probable structural prediction of the protein. In contrast, for the Ebola delta peptide, we could not find any transmembrane domain.

#### 3.2.1. Structural Prediction Using AlphaFold2

From the above assays, it is clear that the glycoprotein CCHF-V-gp exhibits appreciable channel activity and is able to conduct different ionic species. However, until now, no structural model has been available for this membrane protein. Therefore, we employed AlphaFold2 (AF2) [46] to predict the structure and oligomerization state of the protein.

**Figure 2:**
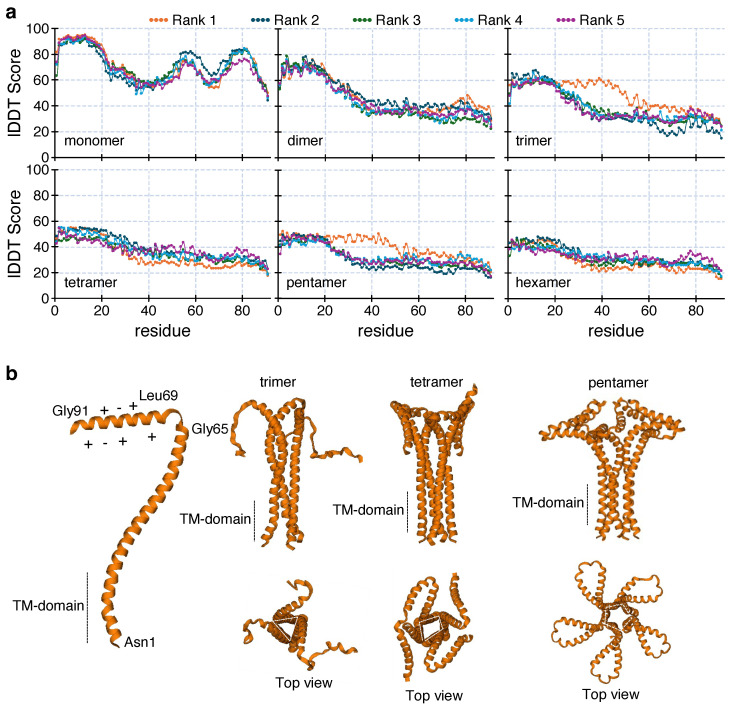
Modeling of CCHF-V-gp. (**a**) lDDT score per residue for predicted oligomers obtained from AlphaFold2-Colab [46]. Five different structural models of different ranks are presented for each oligomer. (**b**) Side and top views of the highest ranking structure of each oligomer.

AlphaFold [77] and its latest predecessor, AlphaFold2 [46], are powerful tools for predicting the 3D structures of protein molecules from their corresponding amino acid sequences. For our membrane ion channel, we checked the structures of a single protein strand and its oligomers (from dimer to hexamer) using the AF2-colab web-based interface [46]. The confidence value was predicted by the local Difference Distance Test (lDDT) score per residue as shown in Figure 2a. A higher lDDT score indicates less deviation in the atomic position of the backbone from the known aligned structures [46,77]. Consequently, the lDDT/residue of different structures for the same oligomer have been predicted with different rankings.

The monomeric unit shows high lDDT per residue for 2–20, 55–60, and 75–82 sequence ranges (more than 90%). However, for other oligomers, the maximum lDDT value drops appreciably. For the dimer, trimer, tetramer, pentamer, and hexamer, the maximum lDDT values are 78%, 67%, 55%, 52%, and 48% respectively. We need to consider a second geometrical constraint before assigning the most probable ion-channel structure. Proteins with a single TM domain can only form a membrane-permeable channel if the degree of oligomerization is three or more [73]. Figure 2b reveals the presence of pore-like geometry for the trimer, tetramer, and pentamer. According to Dai et al., the most common oligomerization states of pore-forming helices are four or five, where the pore sizes optimally act as ion channels [78]. Considering this, we found that between the tetramer and pentamer, the pentameric assembly shows a higher overall sequence-wise confidence value. It is evident from Figure 2a, that the tetrameric assembly has only 20 residues with a comparably high lDDT score (ranging in 48–55%), whereas for the pentamer, around 45 residues have comparable high values (44–52% lDDT range).

Pore formation is a key feature of viral ion channels. The analysis of the pore diameter from the AF2 structures (Table 1), shows suitable pore size for conduction at the top and bottom positions of the transmembrane domain of three different oligomers. To analyze whether these pores form continuous channel structures, we utilized the web-based MOLEonline tool for channel analysis [79]. This tool measures the void space within a protein surface and predicts the possible geometry of pores, cavities, and channels [80].

**Figure 3:**
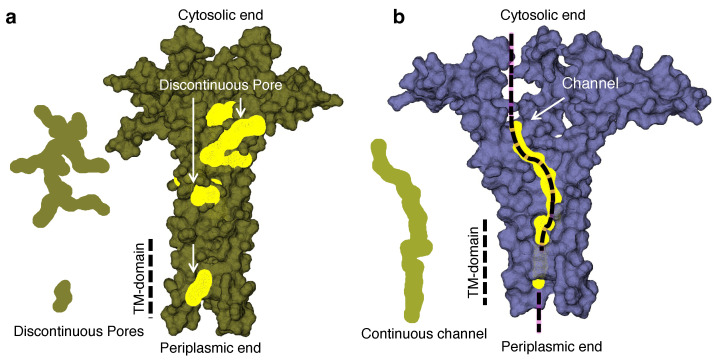
Structure of the pores and continuous channels present in the CCHF-V-gp tetramer (**a**) and pentamer (**b**). The tetramer shows only discrete pore topology, whereas the pentamer has a continuous channel geometry (shown in the left hand side of each structure).

Figure 3 shows the analyzed probable pore/channel structures and their position in the tetramer and pentamer. The tetramer shows no continuous channel across the transmembrane domain; rather, two discrete branched pore structures were found. On the other hand, the pentameric structure exhibited a continuous channel from the periplasmic end, spanning across the transmembrane domain, up to the open end of the cytosolic side.

For the Zaire Ebola virus delta peptide, to predict any structural model with a pore-forming helical bundle within a membrane environment was difficult due to the absence of any transmembrane domain. However, we employed AlphaFold2 to predict the structure of the single peptide strand and compared it with the existing report by He et al. [17] (*vide infra*).

#### 3.2.2. Molecular Dynamic Simulation Studies

Based on the confidence score and channel structure, we considered the pentameric oligomer as the most probable geometry of the protein. To check the viability of the oligomeric state within a membrane environment, we conducted a computer-based molecular dynamics simulation study. The GROMACS package [50,51,52,53,54] was employed as a simulation platform by incorporating the transmembrane domain within an ER-Golgi intermediate compartment (ERGIC)-like membrane.

**Figure 4:**
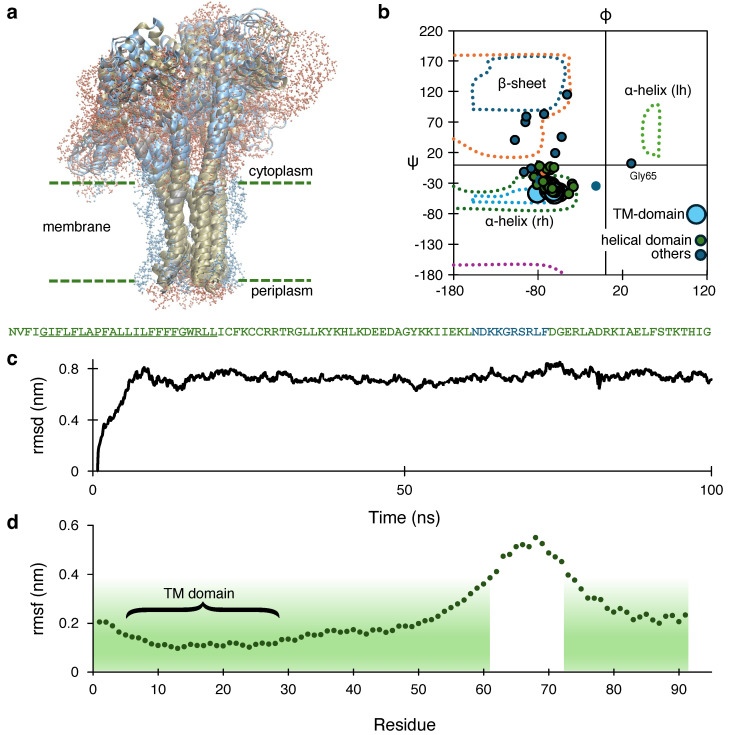
(**a**) MD–simulated structure of the CCHF-V-gp depicting charged residues in blue and hydrophobic residues in yellow. (**b**) Ramachandran plot of amino acid residues indicating the different geometries present in the structure. Each point was obtained by averaging the angles obtained from the molecular dynamics trajectory from 20 to 100 ns. (**c**) RMSD versus time plot of protein backbone atoms over 100 ns trajectory. (**d**) RMSF per residue plot for the protein obtained from the last 50 ns of the trajectory. The transmembrane (TM) domain has been marked within the curve. The green shaded region signifies the helical geometry.

Figure 4 depicts the outcome of a 100 ns simulation of a stable membrane-embedded structure. A plot of the root mean squared deviation (RMSD) of the protein backbone as a function of time reveals a stable backbone for a long range of time, Figure 4c. In particular, the RMSD value of the backbone jumps to 0.8 nm within the first 10 ns and remains almost in the same range for the next 90 ns. The time-averaged Ramachandran plot of different residues (Figure 4b) shows the prevalence of an α-helical structure. Different color coding depicts different regions of a single protein strand: light blue points show the dihedral angles for the TM domain residues, whereas dark green and blue points represent angles of the non-transmembrane helical and non-helical segments, respectively. As expected, the TM domain shows a helical nature, whereby the time-averaged Φ-Ψ angle remains within the right-handed α-helical zone of the Ramachandran plot. However, the other helical zones (residues 28–59 and 60–91) show considerable fluctuation. Figure 4b shows these residues as small blue dots, scattered around non-helical quadrants of the Ramachandran plot.

Based on the above observations, a single protein strand was categorized into three connected segments, as presented in a color-coded format within Figure 4. There are three helical zones from Asn1 to Leu59 (containing the transmembrane domain) and Asp60 to Gly91, colored in green. The root mean squared fluctuation (RMSF) per residue (Figure 4d) corroborates the previous observation. Helical regions (shaded in green) show lower RMSF values than the unstructured region (unshaded zone). Residues spanning the membrane environment (residues 5–27) exhibit low RMSF values due to the constrained environment. After residue Leu27, this value increases, and the region around 65–70 (the unstructured zone), shows the highest range of RMSF values. After residue 70, the fluctuation drops again for the second helical region. Moreover, it is worth mentioning that the second unstructured domain, with the highest fluctuating amino acid residues, forms a turn from Gly65, which is reflected by the unusual position of the residue in the Ramachandran plot.

## 4. Discussion

Crimean–Congo hemorrhagic fever and Ebola fever are deadly infectious diseases, and to date, there is a shortage of effective antiviral drugs available to cure them. Since channels as a family are excellent drug targets, by inference, viral ion channels may represent an attractive target for pharmaceutical point intervention. For example, aminoadamantanes serve as potent anti-influenza agents [32] (widespread resistance not withstanding [33,34]) because they block the virus’s M2 channel [30,31]. Therefore, to achieve this long-term goal, we searched for ion channels from these viruses and characterized their activities using three bacteria-based channel assays.

**Figure 5:**
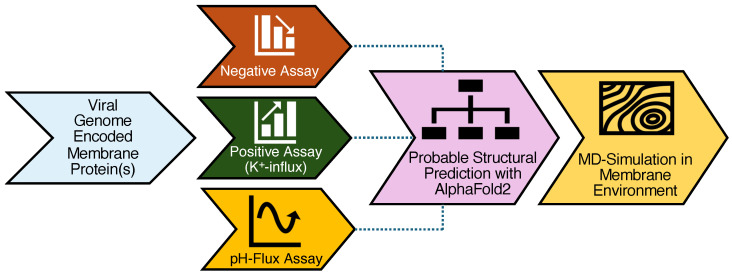
Workflow showing the overall process of identifying and characterizing viroporins employed in the current studies.

Viroporins are small hydrophobic proteins that oligomerize into the host cell membrane and form a hydrophilic pore. These play a major role in viral pathogenicity, mostly by participating in viral particle assembly and release to host cells [28]. Viroporins are mostly TM proteins; therefore, their sequence should contain at least one or more transmembrane domain(s). Such transmembrane domains can be identified with purposefully designed algorithms, such as TMHMM [66]. This TM-domain prediction data coupled with their optimum sequence length (ideally less than 100 amino acids), can be used to identify prospective viroporins. Employing said procedure on the CCHF-V genome, we identified a single protein with 91 amino acids and a single TM domain: CCHF-V-gp. However, the only proteins with TM domains found in the Ebola virus were significantly larger with other known functionalities: The secreted glycoprotein (364 amino acids) plays a major role in host immune evasion [81], while the spike glycoprotein (670 amino acids) mediates the virus’s entry into the host [82]. However, previous reports have identified that the delta peptide can be an effective viroporin [17]. Consequently, we expressed both proteins and examined their activity using the negative, positive, and fluorescence-pH bacteria-based assays. The workflow of our study is summarized in Figure 5.

From the negative assay for the CCHF-V-gp protein, we found a profound reduction in the bacterial growth rate upon protein induction with Isopropyl-β-D-1-thiogalactopyranoside (IPTG). In contrast, minimal decrease in bacterial growth was observed, even at higher IPTG concentrations, upon expression of the Zaire Ebola virus delta peptide (compare Figure 1a and Figure 1d).

A similar picture was obtained in the positive assay, which is reciprocal to the negative assay, in which channel activity causes an increase in bacterial growth. Induction of CCHF-V-gp expression resulted in considerable growth enhancement. In contrast, much higher inducer concentrations were needed to obtain a much smaller impact in the Ebola virus delta peptide (compare Figure 1b and Figure 1e). Notably, increasing the inducer concentration beyond a specific limit (10 μM), while expressing CCHF-V-gp, led to decreased bacterial growth. This phenomenon has been observed in other potent channels, most likely due to excessive membrane permeabilization that offsets the positive impact of K^+^ uptake [83].

The third assay employed measurements of the kinetics of proton influx upon induction of the viral proteins. Once more, appreciable differences were obtained: the expression of CCHF-V-gp led to significant cytoplasmic acidification, while no significant changes were observed upon expression of the Ebola delta peptide (compare Figure 1c and Figure 1f).

Considering the above three bacteria-based assays, it is possible to conclude that the CCHF-V-gp is a potential viroporin: it exhibited activities that are indistinguishable from established channels in every one of the assays [67,68,69,70,72,73,84]. In contrast, the Zaire Ebola Delta peptide could not be designated as a viroporin based on the above methodology since it exhibited poor or lack of activity in all of the assays. Table 2 summarizes the assay results in terms of % activity in each assay to quantify the activities of each channel.

Following structural predictions with AlphFold2 of CCHF-V-gp, the corresponding channel analysis by the MOLEonline revealed that the structure can be pentameric in nature. Although a pentameric structure is plausible based on AlphaFold2 and MOLEonline predictions, alternate stoichiometries cannot be ruled out in the absence of experimental validation. However, considering the “positive-inside” rule [76], we propose a model of CCHF-V-gp, as shown in Figure 4a, with the larger positively charged C-termini inside the cytoplasm. Furthermore, the stability of the model has been examined by molecular dynamics simulation. Specifically, the stability of the ion channel inside the lipid bilayer environment was reflected by a convergence to a stable structure during the course of the simulation (Figure 4c).

**Figure 6:**
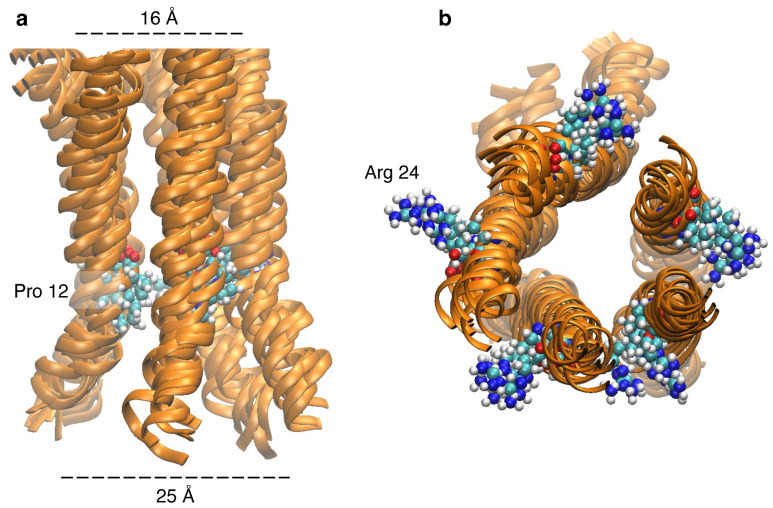
(**a**) Pro12 residue within the transmembrane domain forms a kink in helical bundles to widen the channel structure. (**b**) Transmembrane domain helical bundles from the cytosolic view point, depicting the outwards orientation of Arg24 sidechain.

A closer look at the TM domain of the ion channel reveals that the channel has a wide opening, where the helical units are somewhat tilted outwards due to the presence of Pro12 in the middle of the TM domain (Figure 6a). Moreover, the Arg24 sidechains project outwards to stabilize the negative charges of the phospholipid head groups (Figure 6b). We deduce that these factors, along with the previously discussed structural features, are consistent with the positive experimental results of CCHF-V-gp.

**Figure 7:**
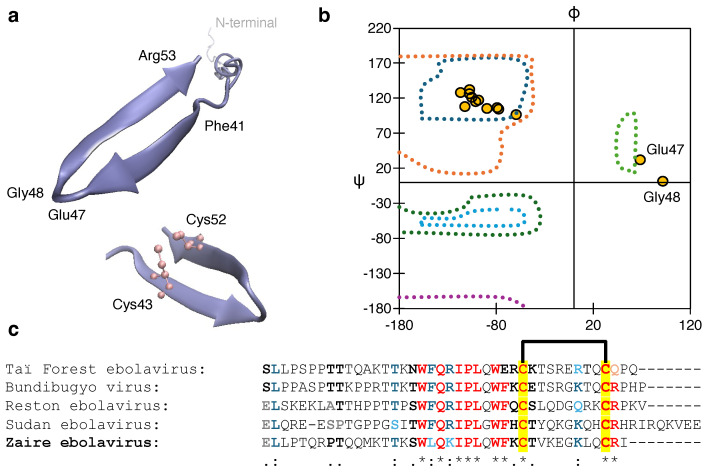
(**a**) β–turn of the Ebola delta peptide C-terminal showing the vicinity of the two Cys residues. (**b**) Ramachandran plot of the β-turn zone showing the dihedral angles. (**c**) Sequence alignment of different Ebola viruses showing the conserved residues in color. The two S-S bond-forming Cys residues are marked in yellow. * (asterisk): All sequences have the exact same amino acid at this position (fully conserved). : (colon): All amino acids at this position have highly similar properties; . (period): Amino acids have moderately similar properties.

We could not find a suitable membrane-bound model for the Zaire Ebola delta peptide, due to the absence of any TM domain predicted by the TM-hidden Markov models. However, He et al. reported that the peptide impacts conductivity in the cell membrane by forming a cyclic structure [17]. Therefore, we checked the AlphaFold structure of the peptide and found a β-turn region in the C-terminus (Figure 7a). The Ramachandran plot (Figure 7b) of the region also verifies this observation, where the residues in the 41 to 53 region show dihedral angles within the twisted β-sheet region. It is also evident from the predicted peptide structure that the two Cys residues at positions 43 and 52 come close, due to the formation of the turned structure, which may also facilitate the S-S bond formation under biological conditions. Interestingly, these two Cys residues are conserved, as observed from the sequence alignment of other types of Ebola viruses shown in (Figure 7c). Despite these facts, our experimental results were unable to observe channel activity in Ebola-delta-peptide. Only a marginal activity was observed at very high IPTG concentration, and our bacteria-based assays are unable to support the designation of the Delta peptide as a viroporin. However, this does not rule out the possibility of its membrane permeabilization. Along with studies reported in reference [17], one more recent finding has reported the membrane-permeabilization nature of Ebola delta peptide [18].

In summary, the glycoprotein (gp) from the Crimean–Congo Hemorrhagic Fever virus (CCHF-V) functions effectively as an ion channel and holds promise as a target for drug development. Both biochemical analyses and structural predictions indicate that CCHF-V-gp is a potential candidate for viroporin designation, and its activity can be checked further in mammalian cells to decipher its intricate physiological role in viral infection. Furthermore, confirmatory methods such as patch clamping can be conducted to check its ion selectivity in the future. In contrast, the Ebola virus delta peptide does not contain an identifiable transmembrane domain, lacks supportive structural predictions, and has not shown significant results in bacteria-based channel assays, making it a less viable target for drug development using channel-blocking assays. However, previous studies have confirmed its membrane permeabilization properties [17,18]. This triggers a scope for future studies for the mechanistic features of such peptides in membrane disruption. Therefore, targeting the CCHF-V-gp viroporin is a promising avenue for developing new antiviral drugs against this deadly hemorrhagic fever.

## Figures and Tables

**Table 1:** Predicted pore diameters for various oligomeric states, along with corresponding maximum lDDT scores of CCHF-V-gp, calculated from AlphFold2 models using MOLEonline.

Oligomeric State	Top (Å)	Bottom (Å)	Average Maximum lDDT Values (%)
Tetramer	27	20	52 (covering 20 residues)
Pentamer	22	24	48 (covering 45 residues)

**Table 2:** Comparison of experimental viroporin activity of CCHF-V-gp and Ebola delta peptide, based on bacterial growth and pH-based assays.

	Activity in Assay (%)		
	Negative	Positive	pH-lux
CCHF-V-gp (ACK58347.1)	69	95	23
Ebola delta peptide (ACK58347.1)	25	9	0

## Data Availability

Data is contained within the article. Further inquiries can be directed to the corresponding author.
